# Autoimmune/inflammatory syndrome induced by buttock augmentation with silicone

**DOI:** 10.1093/rap/rkab034

**Published:** 2021-05-29

**Authors:** Sabreen Ali, Priyanka Mayur Lakhani, Rodney Hughes

**Affiliations:** Rheumatology Department, Ashford and St Peters Hospitals NHS Foundation Trust, Chertsey, UK

A 37-year-old woman presented to the Rheumatology Department with a 3-year history of arthralgia, night sweats and bilateral swollen buttocks. She described unusual levels of fatigue, memory disturbance and low-grade fevers without lymphadenopathy. She had silicone buttock augmentation 10 years earlier. Inflammatory markers, ANA, ENA, ANCA, ACE, immunoglobulins and IgG subclasses were normal; urinalysis was negative. Pelvic MRI demonstrated extensive abnormal signal change, in keeping with silicone deposition within the buttocks, and an inflammatory response extending into the gluteus maximus muscles and surrounding subcutaneous fat. Areas of reduced T2 signal suggested a secondary granulomatous reaction.

There are emerging reports of autoimmune-like phenomena in patients with silicone exposure, coined ASIA syndrome (autoimmune/inflammatory syndrome induced by adjuvants) [1]. Clinical presentation and severity can be heterogeneous.

Silicone may cause an amplified immune response by promoting cytokine release and T-cell proliferation, reacting with mucopolysaccharides in connective tissue and causing granulomatous reactions [1]. Therapeutic options include antibiotics such as minocycline, immunomodulation with CSs or etanercept, and surgical excision. Surgery is limited by the diffuse and extensive nature of the injections, as in this case [2]. Immune reactions to adjuvants can be insidious, and a full medical and cosmetic history should be obtained in patients presenting with autoimmune symptoms.

*Funding*: No funding was received from any bodies in the public, commercial or not-for-profit sectors to carry out this.

*Disclosure statement*: The authors have declared no conflicts of interest.

## Data availability statement

Data are available upon reasonable request by any qualified researchers who engage in rigorous, independent scientific research, and will be provided following review and approval of a research proposal and Statistical Analysis Plan (SAP) and execution of a Data Sharing Agreement (DSA). All data relevant to the study are included in the article.

**Fig. 1:**
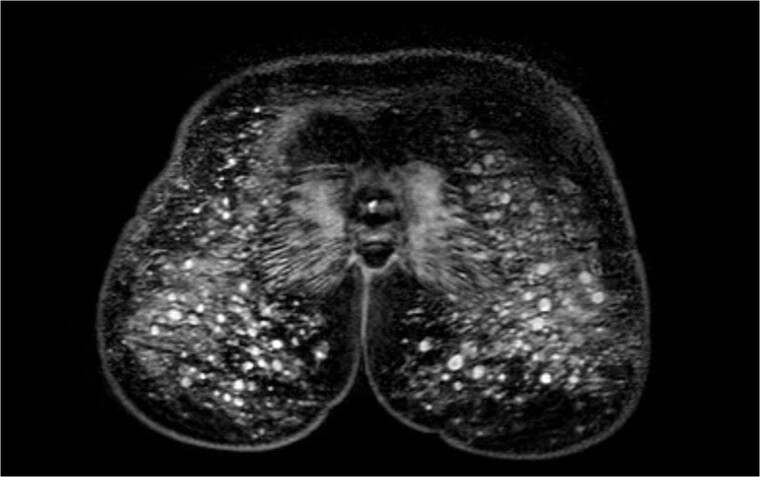
MRI findings: Innumerable rounded lobular intermediate- to low-T1 and intermediate- to high-T2 globules in this patient with ASIA syndrome (autoimmune/inflammatory syndrome induced by adjuvants)

## References

[1] Watad A, Sharif K, Shoenfeld Y. The ASIA syndrome: basic concepts. Mediterr J Rheumatol 2017;28:64–9. 32185259 10.31138/mjr.28.2.64 PMC7046028

[2] Singh M, Solomon I, Calderwood M, Talbot S. Silicone-induced granuloma after buttock augmentation. Plast Reconstr Surg Glob Open 2016;4:e624. 27014553 10.1097/GOX.0000000000000618 PMC4778895

